# Synthesis and Herbicidal Activities of Novel 4-(4-(5-methyl-3-arylisoxazol-4-yl)thiazol-2-yl)piperidyl Carboxamides and Thiocarboxamides

**DOI:** 10.3390/molecules14031288

**Published:** 2009-03-24

**Authors:** De-Jin Hu, Su-Fang Liu, Tong-Hui Huang, Hai-Yang Tu, Ai-Dong Zhang

**Affiliations:** Key Laboratory of Pesticide & Chemical Biology, Ministry of Education, College of Chemistry, Central China Normal University, Wuhan 430079, P. R. China; E-mails: d-eking-h@hotmail.com (D-J.H.), liusufang20062007@163.com (S-F.L.)

**Keywords:** Heterocycles, Piperidine-1-carboxamide, Piperidine-1-thiocarboxamide, D1 protease inhibitor, Herbicide.

## Abstract

A series of novel 4-(4-(5-methyl-3-arylisoxazol-4-yl)thiazol-2-yl)piperidyl carboxamides and thiocarboxamides were synthesized as potential lead compounds of inhibitors targeting D1 protease in plants. These compounds were designed on the basis of a D1 protease inhibitor hit structure identified by homology modeling and virtual screening. The syntheses of these compounds were accomplished via a four-step procedure including the isoxazole ring formation, α-bromination of acetyl group, thiazole ring formation, and carboxamide/thiocarboxamide attachment. The *in vivo* herbicidal activity tests show that most compounds possess moderate to good herbicidal activities. The enzyme activity of one compound against the native spinach D1 protease exhibits a competitive inhibition. The results suggest that these compounds are indeed potential inhibitors for targeting D1 protease in plants.

## 1. Introduction

D1 protease (CtpA) is a carboxyl-terminal processing protease in plants [1], which cleaves the C-terminal extension of the precursor D1 protein (pD1) to form mature D1 protein (mD1). This process is absolutely essential for the assembly of the PSII reaction center [2]. Knockouts of the CtpA gene have resulted in the same non-photoautotrophic phenotype effect as the D1 protein inhibitors applied in plants [3]. Since D1 protease has a high homology in organisms and a lower abundance in plants than D1 protein, it is anticipated that the inhibitors targeting D1 protease may be more efficient than now widely applied inhibitors in targeting D1 protein, a common target protein in current herbicides. At present, however, there is neither a commercial inhibitor, nor any crystal structure information concerning the complex of this enzyme with its substrate precursor D1 protein (pD1) or a potential small molecular inhibitor. Without this information it is difficult to begin the rational design and synthesis of potential inhibitors targeting D1 protease. Recently, Duff and coworkers presented the results of a high throughput screen of available compounds for CtpA protease inhibitors and reported the discovery and inhibitory efficacies of five novel chemical classes of CtpA inhibitors. Evidence suggested that the biological activity of the compounds might be at least partially due to their inhibition of CtpA [4].

**Figure 1:**
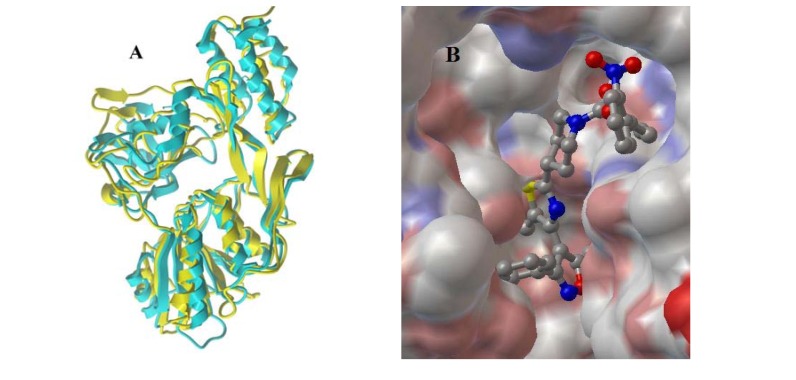
A) the alignment of the D1 protease model of spinach with the template **1cf6**; B) the docking pattern of the lead hit **T06126** into the active site of spinach D1 protease model.

In our earlier work, we built the D1 protease model of a higher plant (spinach) with the homology modeling program Modeller9v6 and model checking program PROCHECK, on the basis of the template **1cf6**, which is a three-dimensional X-ray crystal structure of the D1 protease from the green alga* Scenedesmus obliquus* [5]. The alignment of the model with the template exhibited a good match, as shown in Figure 1A.

The virtual screening of a series of self-built and commercial compound libraries was accomplished by docking compounds into the active site of the spinach D1 protease model with the program FlexX. The top-scoring compounds were further examined visually. A lead hit, 4-(4-(5-methyl-3-phenylisoxazol-4-yl)thiazol-2-yl)piperidine-1-carboxamide (**T-06162**, from the compound libraries Lead 3&4, Maybridge), was identified as having expected inhibitive efficacy. The docking pattern of this lead hit compound in the active site of spinach D1 protease model is shown in Figure 1B. This docking was accomplished with the docking programs Autogrid4 and Autodock4 via the graphical user interface tool Autodochtools. The predicted binding energy and inhibition constant *Ki* were -14.56 Kcal/mol and 20.81 pM, respectively. 

**Scheme 1:**
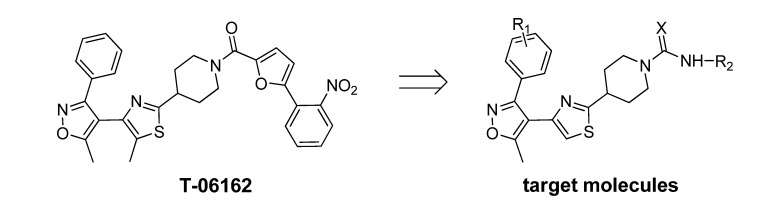
The design of the target molecules.

**Scheme 2:**
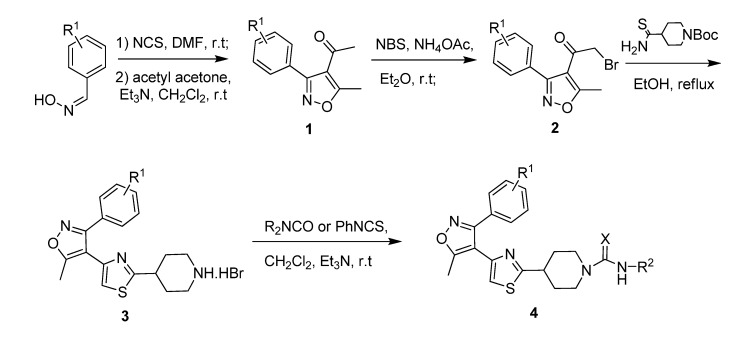
Synthetic pathway for preparation of the target compounds.

Based on this lead hit information, a series of novel (4-(5-methyl-3-phenylisoxazol-4-yl)thiazoles (Scheme 1) containing piperidine-1-carboxamide or piperidine-1-thiocarboxamide moiety were designed for chemical synthesis and biological testing. The synthesis was accomplished via a four-step procedure including the isoxazole ring formation, α-bromination of the acetyl group, thiazole ring formation, and carboxamide/thiocarboxamide attachment (Scheme 2), to obtain the target compounds. The *in vivo* herbicidal efficacies of these compounds were tested against two model plants, *Brassica napus* (rape) and *Echinochloa crusgalli* (barnyard grass),and most of them possessed moderate to good herbicidal activities. As a preliminary lead compound validation, one of the synthesized compounds was tested for its enzyme inhibitive efficacy against the native spinach D1 protease with a HPLC assay method, by employing a mimic polypeptide substrate S24. The S24 has a sequence identical to the C-terminal of the precursor D1 protein [6]. 

## 2. Results and Discussion

### 2.1. Chemistry

All title compounds were synthesized via a four-step procedure (Scheme 2). The key intermediate, 4-acetyl-5-methyl-3-arylisoxazole **1**, was prepared according to the procedure described in the literature, with some modifications [7]. The oxime chloride, which was transformed into the corresponding nitrile oxide *in situ* by base treatment, was added to 2 equivalents of acetyl acetone in dichloromethane using triethylamine as base to give the isoxazole **1**. *N*-bromosuccinimide was used as brominating reagent to easily form the α-bromo ketone **2** [8], along with a trace of polybromominated derivative. The mixture without purification was refluxed with 4-thiocarbamoylpiperidine-1-carboxylic acid tert-butyl ester, which was prepared as a published procedure [9], to obtain the intermediate 4-[4-(3-aryl-5-methylisoxazol-4-yl)thiazol-2-yl]piperidine hydrobromide salt **3** [10], where the protected group Boc was removed with hydrobromide formed *in situ*. The amine group of the piperidine in the intermediate **3** was released with triethylamine and subjected to react with isocyanate or thioisocyanate to afford the carboxamide/thiocarboxamide compound **4**. 

**Figure 2:**
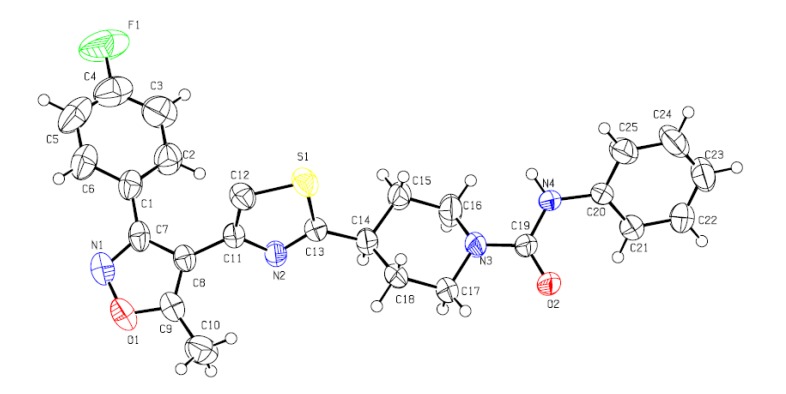
Molecular structure of compound** 4j**.

The molecular structures of the title compounds were deduced from their spectra data (IR, ^1^H-NMR, ^13^C-NMR, LC-MS) and elemental analyses. The structure of compound **4j** was also confirmed by the result of a single crystal X-ray structure determination (Figure 2). 

Experimental details for data collection and structure refinement are summarized in Table 1. The characteristic ^1^H-NMR chemical shift values of the carboxamide/thiocarboxamide amido N-Hs and the protons at positions 2 and 6 on the piperidine ring are significantly affected by the carbonyl or thiocarbonyl group. The characteristic ^1^H-singlets of the *N*-*p*-fluorophenylcarboxamide N-Hs in compounds **4c~4g** appear in the region of 6.92~6.44 ppm, whereas that of the *N*-phenyl-thiocarboxamide N-Hs in compounds **4k, 4o** appear in the region of 7.32~7.24 ppm. For the *N*-isopropyl- or *N*-butylcarboxamide compounds **4h, 4i, 4l, 4m**, the characteristic ^1^H-signlets of the active amido N-Hs are located in the region of 4.25~4.50 ppm. Meanwhile, the ^1^H peaks of the piperidine rings are complicated, but still can be clearly assigned. There are five sets of proton peaks because the piperidine ring adopts a chair conformation. The chemical shift values of the two sets, axial H2a-H6a and exponential H2e-H6e, are differently affected by the carboxamide or thiocarboxamide and the *N*-substituent, attributive to the neighboring interaction of protons at position 2 and 6 with the carbonyl or thiocarbonyl group. For example, the H2a-H6a set appears as a doublet and has a chemical shift at ~3.95 ppm for *N*-alkylcarboxamides **4h, 4i, 4l, 4m**, ~4.12 ppm for *N*-arylcarboxamides **4a~4g**, **4j, 4n**, and ~4.46 ppm for thiocarboxamides **4k, 4o**, respectively. The exponential H2e-H6e sets do not show large differences in chemical shifts in these compounds.

**Table 1:** Summary of crystallographic data and parameters of compound **4j**.

Empirical formula	C_25_H_22_FN_4_O_2_S
Formula weight	461.53
Temperature	298(2) K
Wavelength	0.71073 Å
Crystal system	Orthorhombic
Space group	Pbca
Unit cell dimensions	a= 10.0701(7) Å α= 90^o^
	b= 11.1317(8) Å β= 90^o^
	c= 41.558(3) Å γ= 90^o^
Volume	4658.6(6) Å^3^
Z	8
Density (calculated)	1.316 Mg/m^3^
Absorption coefficient	0.177 mm^-1^
F(000)	1928
Crystal size	0.23 x 0.16 x 0.10 mm^3^
Theta range for data collection	0.98 to 26.00°.
Index ranges	-11<=h<=12, -13<=k<=13, 51<=l<=51
Reflections collected	28193
Independent reflections	4584 [R(int) = 0.0820]
Completeness to theta = 26.00	100.0 %
Absorption correction	None
Max. and min. transmission	0.9826 and 0.9605
Refinement method	Full-matrix least-squares on F^2^
Data / restraints / parameters	4584 / 1 / 305
Goodness-of-fit on F^2^	0.917
Final R indices [I>2sigma(I)]	R1 = 0.0515, wR2 = 0.1219
R indices (all data)	R1 = 0.0878, wR2 = 0.1343
Largest diff. peak and hole	0.562 and -0.222 e^-3^

### 2.2. Biological Activities

The herbicidal activities of all compounds **4a~4o** against *Brassica napus* (rape) and *Echinochloa crusgalli* (barnyard grass) have been investigated at dosages of 100 mg/L and 10 mg/L using the known procedure [11], and compared with distilled water as the control. The bioassay results show that most of the synthesized compounds possess moderate to good herbicidal activities when fluorine-containing phenyl groups are introduced into molecular structures. The inhibition rates are listed in Table 2.

**Table 2:**
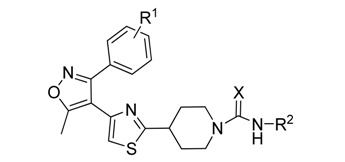
Herbicidal activity of the target compounds **4a**~**4o** and their logP values.

Comp.	R^1^	R^2^	X	Relative inhibition (root %/stalk %)				clogP
				Rape		Barnyard grass		
				100 mg/L	10 mg/L	100 mg/L	10 mg/L	
**4a**	H	Phenyl	O	80.4/70.0	51.4/30.0	78.3/63.6	52.0/52.3	4.33
**4b**	4-OCH_3_	Phenyl	O	43.8/4.5	20.2/-22.7	76.1/24.6	45.7/-22.0	4.38
**4c**	H	4-F-phenyl	O	85.4/31.8	66.3/0	87.1/17.7	54.8/0	4.49
**4d**	4-OCH_3_	4-F-phenyl	O	81.3/53.6	46.9/14.3	86.4/37.0	59.1/34.8	4.55
**4e**	4-F	4-F-phenyl	O	76.1/56.7	35.8/6.7	74.2/52.3	35.5/50.0	4.66
**4f**	2-F	4-F-phenyl	O	96.6/66.7	52.3/16.7	80.6/63.6	64.5/56.8	4.61
**4g**	2,4-diCl	4-F-phenyl	O	90.3/70.0	34.9/20.2	83.8/63.6	32.3/45.5	5.77
**4h**	4-F	*i*Pr	O	87.2/66.7	68.8/46.7	83.9/63.6	54.8/47.7	4.02
**4i**	4-F	Bu	O	88.1/70.0	67.8/40.0	87.1/63.6	71.0/56.8	4.78
**4j**	4-F	Phenyl	O	83.3/53.6	46.9/25.0	95.5/56.5	65.9/39.1	4.49
**4k**	4-F	Phenyl	S	90.1/53.3	54.1/6.7	87.1/63.6	48.4/43.2	4.40
**4l**	2-F	*i*Pr	O	83.1/54.5	68.5/22.7	73.9/14.6	56.5/22.0	3.97
**4m**	2-F	Bu	O	86.2/70.0	61.5/43.3	90.3/63.6	58.1/61.4	4.74
**4n**	2-F	Phenyl	O	91.2/72.7	56.2/31.8	84.8/39.0	56.5/17.1	4.46
**4o**	2-F	Phenyl	S	92.7/60.0	64.2/43.3	83.9/59.1	48.4/45.5	4.35

The preliminarily discussion of the structure-bioactivity relationships of these compounds is as follows: from Table 2, it can be seen that all compound have moderate to high herbicidal activities at a high concentration (100 mg/L). For example, **4f**, **4g**, **4k**, **4n**, **4o** show inhibitory rates larger than 90% against the root growth of rape at 100 mg/L. However, when the applied concentration is decreased to 10 mg/L, the inhibitive efficacy obviously decreases. Introduction of fluorine-containing phenyl group to the isoxazole moiety obviously influences the herbicidal activity in some cases, and its relative position (*o-* or *p*-F) on the benzene ring plays an important role. The compounds **4f**, **4n** with *ortho*-fluoro on the phenyl ring have higher activities, whereas the compounds **4e**, **4j** with *para*-fluoro on the phenyl ring obviously have lower activities, even lower than that of the corresponding non-fluorine containing compounds **4a**, **4b**. On the other hand, the chlorine-containing compound **4g** also shows good activity. It is worth mention that there is no obvious difference in activities between carboxamides and thiocarboxamides.

According the calculation results of octanol/water partition coefficients for these synthesized compounds with the online Molinspiration LogP calculation program [12], the clogP values are ranged from 4 to 5 (Table 2), higher than that of common herbicides (whose values are usually less than 3, for example, the value of the widely used herbicide atrazine is 2.55). An interesting finding is that when the applied concentrations are decreased for compounds with low clogP values, the herbicidal activities will be decreased to a less extent, compared with that for compounds with higher clogP values. For example, the compound **4l**, which has a logP value 3.97, the herbicidal efficacy declined by 15 percent when the applied concentration is decreased from 100 mg/L to 10 mg/L. For compound **4f** with a clogP 5.77, however, the herbicidal efficacy dropped by 55% under the same conditions. This may imply that lipophilicity plays an important role in the bioavailability for the title compounds.

**Figure 3:**
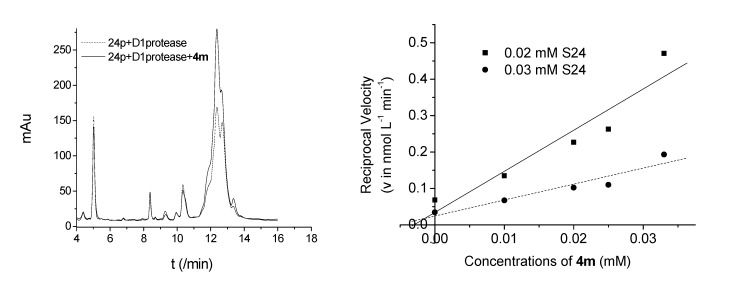
A) The HPLC elution profiles of S24 before (dotted line) and after (solid line) the addition of **4m**. B) the Dixon plot for determine inhibition constant *Ki*.

The inhibitive efficacy of compound **4m** against the native spinach D1 protease was assayed via a HPLC assay method by employing the polypeptide S24 as the substrate mimic. The typical HPLC elution profiles of S24 before (dotted line) and after (solid line) the addition of compound **4m** are shown in Figure 3A. With the addition of compound **4m**, the S24 content (retention time 12.37 min) obviously increases, whereas the content of the hydrolytic product S9 (retention time 5.02 min) decreases at the same time, comparing with the result in the case without the addition of compound **4m**. The inhibition type and inhibition constant (*Ki*) can be determined by the double-reciprocal plots of enzyme kinetics (data not shown) and the Dixon method [13], respectively (Figure 3B). The inhibition behaves as a competitive type, and the inhibition constant *Ki* is estimated to be 1.3 μM. This means there is a direct inhibition effect of **4m** against D1 protease. Since the structural similarity, it is reasonable that these synthesized compounds have inhibitive effect to D1 protease and may be employed as lead compounds for the development of D1 protease inhibitors.

## 3. Experimental

### 3.1. General

IR spectra were determined on a Bruker TENSOR 27 infrared spectrometer as KBr film with absorption in cm^-1^. ^1^H-NMR spectra were recorded on a Mercury Plus-600 (600 MHz) spectrophotometer. Chemical shifts were reported in ppm with TMS as the internal standard. ^13^C-NMR spectra were also recorded on this spectrophotometer. Mass spectra were obtained on an API 2000 liquid chromatography-tandem mass spectrometer. Elemental analyses were taken on a Vario EL III elemental analyzer. Single crystal X-ray data was collected on a Bruker Smart Apex CCD detector.

### 3.2. Crystallographic Data

CCDC 721766 contains the supplementary crystallographic data for this paper. These data can be obtained free of charge via www.ccdc.cam.ac.uk/conts/retrieving.html (or from the CCDC, 12 Union Road, Cambridge CB2 1EZ, UK; Fax: +44 1223 336033; e-mail: deposit@ccdc.cam.ac.uk).

### 3.2. In vivo Herbicidal Activity

The *in vivo* herbicidal activity was examined for the growth inhibition effect of the dicotyledon rape *Brassica napus* and the monocotyledon barnyardgrass *Echinochloa crusgalli*, with the standard Petri dish test and the pre-emergence treatment procedure. Briefly, the compounds to be tested were dissolved in DMF and emulsified with Tween^®^ 80, and the solutions were diluted with water to the concentrations of 100 μg/mL and 10 μg/mL, respectively. Nine mL of the solution was added in a Petri dish and two pieces of filter paper were placed on the dish bottom. Twenty seeds of each one of rape and barnyard grass were placed on the filter paper. The covered Petri dish was transferred into an artificial climate incubator (MGC-400H, Shantou Keyi Co., Ltd., P.R. China), where the condition was controlled at temperature 23 ± 1 °C, room humidity 60 ± 5%, light intensity 10 Klux, and photoperiod 12 h/day. The incubation was continued for 5 days. The lengths of 10 roots and 10 stems were measured and the means were calculated. The percentage inhibition was calculated relative to controls using distilled water instead of the compound solution.

### 3.3. Enzyme Activity

The native spinach CtpA protein was purified from spinach thylakoid with the method described [14], with slight modifications. The modifications included the separation of the tissue residue with centrifuge instead of filtration with cheesecloth and Mirracloth in the extraction procedure and the use of the GE healthcare ÄKTA purifier in the purification procedure through an Q. FF anion exchange column and a Superdex 75 HR 10/30 column instead of the Bio-Rad UNO Q-6R system. The final CtpA had a purity of larger than 90% with a specific activity 1.7 pm/min/mg.

The mimic substrate S24 of CtpA, a 24-mer oligopeptide corresponding to the C-terminal sequence of the D1 precursor protein, deduced from the nucleotide sequence of spinach psbA gene [15], was synthesized by GL Biochem (Shanghai) Ltd, P.R. China. S24 had a purity of 95.0% with a molecular weight found 868.25 (M+3H^+^)^3+^, calculated 2604.91 (C_112_H_174_N_34_O_36_S). The sequence is listed as the following: VMHERNAHNFPLDLA*AIEAPSTNG, where the asterisk labeled position indicates the proteolytic positon cleavage by CtpA.

For the enzyme activity measurement, the tested compound was dissolved in the buffer solution (50 mM HEPES, pH 7.7, 100 mM NaCl, 100 mL/L glycerol, 0.5 g/L Triton^®^ X-100) at different concentrations of 0, 10, 20, 25, 33 μM, respectively. A suitable amount of D1 protease in buffer solution was added and the solution was kept at 25 °C for 30 minutes, and then an indicated amount of the mimic substrate S24 oligopeptide was added. In a typical experiment, the enzymatic reaction was carried out at 25 ºC for 6 h. The reaction was terminated by the addition of 20 μL trifluoroacetic acid solution (0.1 M in the buffer) and then the mixture was centrifuged at 12,000 r.min^-1^ for 10 minutes. An aliquot (20 μL) of the resultant supernatant solution was subjected to HPLC analysis using an Agilent system consisting of a C8 reverse phase column. Two solutions were combined for elution: Solution A, 0.1% (v/v) TFA/water; Solution B, 0.1% (v/v) TFA/acetonitrile. Elution was conducted with a linear gradient (solution A: from 90% to 60%, solution B: 10% to 40%) in 20 minutes. Elution was monitored at 220 nm. The average reaction rates were calculated according to the increase of the integral area of S24 before and after the addition of the compound. The inhibition type was determined via the double-reciprocal plots of enzyme kinetics and inhibition constant (*Ki*) was determined by the Dixon method [13]. All graphs were plotted and fitted using Origin 7.0 (OriginLab Corp., Northampton, MA, USA).

### 3.4. General Method for the Preparation of 4-Acetyl-5-methyl-3-aryl-isoxazoles **2a~2e**

Oxime (10 mmol) was dissolved in DMF (30 mL), cooled with ice-water bath. To the solution, NCS (12 mmol) was added in portions. After the addition, the mixture was stirred at room temperature for 4~6 h. Then ethyl acetate (30 mL) was added, and the organic phase was washed with 50% NaCl (2 × 30 mL) and water (2 × 30 mL). The organic phase was dried with anhydrous Na_2_SO_4_ and concentrated under vacuum. The residue was dissolved in CH_2_Cl_2_ (30 mL), and acetylacetone (20 mmol) was added. The mixture cooled in an ice-water bath and Et_3_N (12 mmol) was added dropwise. After of the addition, the reaction was continued for additional 2~4 h at room temperature. TLC was used to indicate the end of reaction. The mixture was washed with water, dried with Na_2_SO_4_, and concentrated in vacuum. The desired product was obtained by purification with recrystallization from the mixed solvent of ethyl acetate and petroleum ether or column chromatography on silica gel. 

*4-Acetyl-5-methyl-3-phenylisoxazole* (**1a**): white solid, yield 57%, mp 145~146 ºC (lit [7]: 145~147 ºC).

*4-Acetyl-3-(4-methoxyphenyl)-5-methylisoxazole* (**1b**): white solid, yield 61%, mp 56~58 ºC (lit [7]: 55~57 ºC).

*4-Acetyl-3-(4-fluorophenyl)-5-methylisoxazole*** (1c**): thick orange oil, yield 65%; ^1^H-NMR (CDCl_3_,) δ: 7.51 (m, 2H, ArH), 7.18 (m, 2H, ArH), 2.72 (s, 3H, CH_3_), 2.15 (s, 3H, COCH_3_).

*4-Acetyl-3-(2-fluorophenyl)-5-methylisoxazole* (**1d**): thick yellow oil, yield 74%; ^1^H-NMR (CDCl_3_) δ: 7.52 (m, 2H, ArH), 7.28 (m, 1H, ArH), 7.20 (m, 1H, ArH), 2.72 (s, 3H, CH_3_), 2.15 (s, 3H, COCH_3_).

*4-Acetyl-3-(2,4-dichlorophenyl)-5-methylisoxazole* (**1e)**: thick orange oil, yield 73%;^ 1^H-NMR (CDCl_3_) δ: 7.55 (s, 1H, ArH), 7.40 (m, 2H, ArH), 2.72 (s, 3H, CH_3_), 2.15 (s, 3H, COCH_3_).

### 3.5. General Method for the Preparation of 4-(4-(5-methyl-3-arylisoxazol-4-yl)thiazol-2-yl)piperidyl Carboxamides and Thiocarboxamides **4a~4o**

To a mixture of ketone **1a~1e** (1 mmol) and NH_4_OAc (0.1 mmol) in Et_2_O (5 mL) NBS (1.1 mmol) was added. The mixture was stirred at room temperature for 30 min to 1 hour. The mixture was filtered and the filtrate was washed with water, dried with Na_2_SO_4_ and concentrated under vacuum. The residue contained the major product α-monobromo ketone **2** and a trace of α-polybrominated byproduct. The residue was used directly for the following reactions without further purification.

The following procedure was employed for the preparation of intermediate **3**, according to the reported method [10]. The above α-monobromo ketone **2 **(ca. 1 mmol) was dissolved in EtOH (15 mL), and 4-thiocarbamoylpiperidine-1-carboxylic acid *tert*-butyl ester [9] (1.1 mmol) was added. The mixture was heated to reflux for 2 hours, then cooled to room temperature, and poured into Et_2_O with stirring. The salts** 3a~3e** were participated, filtered, dried under vacuum, and used without further purification.

The salt **3** (ca. 1 mmol) was dissolved in CH_2_Cl_2_ (10 mL) containing Et_3_N (2 mmol), then the appropriate isocyanate or phenylisothiocyanate (1.05 mmol) was added dropwise at room temperature. When the reaction completed (about 10 min), solvent was evaporated, and the residue was purified by column chromatography on silica gel (CH_2_Cl_2_-petroleum ether, 5:1) to afford the desired products** 4a~4o**.

*N-phenyl-4-(4-(5-methyl-3-phenylisoxazol-4-yl)thiazol-2-yl)piperidine-1-carboxamide* (**4a**). White solid, mp 108.3~110.1 ºC, yield 95%. IR (KBr) ν: 3314, 1635(C=O), 1598, 1531 cm^-1^; ^1^H-NMR (CDCl_3_) δ: 7.51 (m, 2H, ArH), 7.44~7.29(m, 7H, ArH), 7.04 (m, 1H, ArH), 6.78 (s, 1H, thiazole 2-H), 6.41 (s, 1H, NHCO), 4.13 (q, 2H, piperidine H2a, H6a), 3.23 (m, 1H, CH), 3.10 (m, 2H, piperidine H2e, H6e), 2.65 (s, 3H, CH_3_), 2.18 (m, 2H, piperidine H3a, H5a), 1.88 (m, 2H, piperidine H3e, H5e); ^13^C-NMR (CDCl_3_) δ: 173.60, 168.18, 161.38, 154.90, 144.66, 138.96, 129.55, 129.14, 128.80, 128.65, 128.46, 123.02, 119.95, 115.33, 110.46, 43.93, 40.11, 31.95, 12.49; LC-MS m/z: 444.5 (M), 466.4 (M + Na^+^). Elemental Anal. calcd. for C_25_H_24_N_4_O_2_S: C, 67.54; H, 5.44; N, 12.60; Found C, 67.59; H, 5.35; N, 12.55.

*N-phenyl-4-(4-(3-(4-methoxyphenyl)-5-methyl-4-yl)thiazol-2-yl)piperidine-1-carboxamide* (**4b**). White solid, mp 129.5~130.6 ºC, yield 90%. IR (KBr) ν: 3327, 1640 (C=O), 1596, 1534 cm^-1^; ^1^H-NMR (CDCl_3_) δ: 7.44 (m, 2H, ArH), 7.34 (d, *J*=7.8 Hz, 2H, ArH), 7.26 (m, 2H, ArH), 7.00 (m, 1H, ArH), 6.89 (m, 2H, ArH), 6.82 (s, 1H, thiazole 2-H), 6.81(bs, 1H, NHCO), 4.12 (d, 2H, piperidine H2a, H6a), 3.81 (s, 3H, OCH_3_), 3.18 (m, 1H, CH), 3.02 (m, 2H, piperidine H2e, H6e), 2.60 (s, 3H, CH_3_), 2.14 (m, 2H, piperidine H3a, H5a), 1.83 (m, 2H, piperidine H3e, H5e); ^13^C-NMR (CDCl_3_) δ: 173.54, 168.00, 160.97, 160.51, 155.10, 144.79, 129.91, 128.68, 122.90, 121.33, 120.02, 115.34, 113.84, 110.34, 55.19, 43.89, 40.11, 31.95, 12.38; LC-MS m/z: 474.4 (M), 496.2 (M + Na^+^); Elemental Anal. calcd. for C_26_H_26_N_4_O_3_S: C, 65.80; H, 5.52; N, 11.81; Found C, 65.85; H, 5.51; N, 11.77.

*N-(4-fluorophenyl)-4-(4-(5-methyl-3-phenylisoxazol-4-yl)thiazol-2-yl)piperidine-1-carboxamide* (**4c**). White solid, mp 124.3~125.8 ºC, yield 89%. IR (KBr) ν: 3245 (N-H), 1633 (C=O), 1528, 1511, 1504, 1214 (C-F) cm^-1^; ^1^H-NMR (CDCl_3_) δ: 7.51 (t, *J*=7.2 Hz, 2H, ArH), 7.29 (m, 2H, ArH), 7.41 (m, 3H, ArH), 6.95 (m, 2H, ArH), 6.94 (s, 1H, NHCO), 6.83 (s, 1H, thiazole 2-H), 3.99 (d, 2H, piperidine H2a, H6a), 3.17 (m, 1H, CH), 2.95 (t, 2H, piperidine H2e, H6e), 2.66 (s, 3H, CH_3_), 2.12 (d, 2H, piperidine H3a, H5a), 1.80 (m, 2H, piperidine H3e, H5e); ^13^C-NMR (CDCl_3_) δ: 173.51, 168.20, 161.41, 160.05, 157.64, 154.97, 144.73, 134.82, 129.57, 128.67, 122.03, 115.35, 110.47, 43.93, 40.11, 31.95, 12.49; LC-MS m/z: 462.1 (M), 484.4 (M + Na^+^); Elemental Anal. calcd. for C_25_H_23_FN_4_O_2_S: C, 64.92; H, 5.01; N, 12.11; Found C, 64.95; H, 5.07; N, 12.18.

*N-(4-fluorophenyl)-4-(4-(3-(4-methoxyphenyl)-5-methylisoxazol-4-yl)thiazol-2-yl)piperidine-1-carbox-amide* (**4d**). White solid, mp 171.0~172.5 ºC, yield 85%. IR (KBr) ν: 3234 (N-H), 1632 (C=O), 1533, 1509, 1212 (C-F) cm^-1^; ^1^H-NMR (CDCl_3_) δ: 7.43 (m, 2H, ArH), 7.27 (m, 2H, ArH), 6.94~6.92 (m, 4H, ArH), 6.88 (s, 1H, NHCO), 6.82 (s, 1H, thiazole 2-H), 4.12 (d, 2H, piperidine H2a, H6a), 3.81 (s, 3H, OCH_3_), 3.20 (m, 1H, CH), 3.02 (m, 2H, piperidine H2e, H6e), 2.60 (s, 3H, CH_3_), 2.14 (m, 2H, piperidine H3a, H5a), 1.83 (m, 2H, piperidine H3e, H5e); ^13^C-NMR (CDCl_3_) δ: 173.54, 168.03, 161.01, 155.10, 144.80, 129.91, 122.07, 121.31, 115.28, 113.85, 110.36, 55.19, 43.85, 40.09, 31.94, 12.38; LC-MS m/z: 492.4 (M), 513.8 (M + Na^+^); Elemental Anal. calcd. for C_26_H_25_FN_4_O_3_S: C, 63.40; H, 5.12; N, 11.37; Found: C, 63.45; H, 5.14; N, 11.41. 

*N-(4-fluorophenyl)-4-(4-(3-(4-fluorophenyl)-5-methylisoxazol-4-yl)thiazol-2-yl)piperidine-1-carbox-amide* (**4e**). White solid, mp 147.1~148.9 ºC, yield 88%. IR (KBr) ν: 3267 (N-H), 1634 (C=O), 1610, 1512, 1218 (C-F) cm^-1^; ^1^H-NMR (CDCl_3_) δ: 7.51 (m, 2H, ArH), 7.27 (m, 2H, ArH), 7.08 (m, 2H, ArH), 6.94 (m, 2H, ArH), 6.84 (s, 1H, thiazole 2-H), 6.76 (s, 1H, NHCO), 4.12 (d, 2H, piperidine H2a, H6a), 3.21 (m, 1H, CH), 3.02 (m, 2H, piperidine H2e, H6e), 2.61 (s, 3H, CH_3_), 2.14 (m, 2H, piperidine H3a, H5a), 1.82 (m, 2H, piperidine H3e, H5e); ^13^C-NMR (CDCl_3_) δ: 173.80, 168.36, 164.29, 162.64, 160.47, 159.62, 158.01, 155.11, 144.53, 134.93, 130.60, 125.24, 122.21, 115.35, 110.44, 43.90, 40.15, 31.94, 12.35; LC-MS m/z: 480.6 (M), 502.1 (M + Na^+^); Elemental Anal. calcd. for C_25_H_22_F_2_N_4_O_2_S: C, 62.49; H, 4.61; N, 11.66; Found: C, 62.46; H, 4.68; N, 11.70.

*N-(4-fluorophenyl)-4-(4-(3-(2-fluorophenyl)-5-methylisoxazol-4-yl)thiazol-2-yl)piperidine-1-carbox-amide* (**4f**). White solid, mp 79.2~81.2 ºC, yield 88%. IR (KBr) ν: 3289 (N-H), 1631 (C=O), 1536, 1510, 1222 (C-F) cm^-1^; ^1^H-NMR (CDCl_3_) δ: 7.51~7.45 (m, 2H, ArH), 7.49~7.27 (m, 3H, ArH), 7.10 (m, 1H, ArH), 6.97 (m, 2H, ArH), 6.71 (s, 1H, thiazole 2-H), 6.51 (s, 1H, NHCO), 4.06 (d, 2H, piperidine H2a, H6a), 3.19 (m, 1H, CH), 3.07 (m, 2H, piperidine H2e, H6e), 2.73 (s, 3H, CH_3_), 2.13 (m, 2H, piperidine H3a, H5a), 1.82 (m, 2H, piperidine H3e, H5e); ^13^C-NMR (CDCl_3_) δ:174.03, 168.06, 164.31, 162.70, 161.39, 158.80, 157.54, 144.66, 131.58, 124.35, 120.21, 117.73, 116.02, 113.46, 110.66, 43.72, 40.13, 31.88, 12.47; LC-MS m/z: 480.5 (M), 502.3 (M + Na^+^); Elemental Anal. calcd. for C_25_H_22_F_2_N_4_O_2_S: C, 62.49; H, 4.61; N, 11.66; Found: C, 62.52; H, 4.65; N, 11.71.

*N-(4-fluorophenyl)-4-(4-(3-(2,4-dichlorophenyl)-5-methylisoxazol-4-yl)thiazol-2-yl)piperidine-1-carboxamide* (**4g**). White solid, mp 147.3~148.7 ºC, yield 81%. IR (KBr) ν: 3277 (N-H), 1635 (C=O), 1547, 1510, 1214 (C-F), 726 (C-Cl) cm^-1^; ^1^H-NMR (CDCl_3_) δ: 7.49 (s, 1H, ArH), 7.40 (m, 2H, ArH), 7.28 (m, 2H, ArH), 6.99 (m, 2H, ArH), 6.59 (s, 1H, thiazole 2-H), 6.44 (s, 1H, NHCO), 4.06 (m, 2H, piperidine H2a, H6a), 3.19 (m, 1H, CH), 3.08 (m, 2H, piperidine H2e, H6e), 2.76 (s, 3H, CH_3_), 2.12 (m, 2H, piperidine H3a, H5a), 1.80 (m, 2H, piperidine H3e, H5e); ^13^C-NMR (CDCl_3_) δ: 173.37, 167.77, 160.07, 159.15, 157.66, 154.92, 144.53, 136.32, 134.85, 132.34, 129.73, 127.57, 121.95, 115.43, 113.30, 111.69, 43.84, 39.89, 31.82, 12.94; LC-MS m/z: 531.0 (M), 552.7 (M + Na^+^); Elemental Anal. calcd. for C_25_H_21_Cl_2_FN_4_O_2_S: C, 56.50; H, 3.98; N, 10.54; Found: C, 56.47; H, 3.92; N, 10.57.

*N-isopropyl-4-(4-(3-(4-fluorophenyl)-5-methylisoxazol-4-yl)thiazol-2-yl)piperidine-1-carboxamide* (**4h**). White solid, mp 140.5~141.2 ºC, yield 91%. IR (KBr) ν: 3354 (N-H), 1616 (C=O), 1548, 1458, 1224 (C-F) cm^-1^; ^1^H-NMR (CDCl_3_) δ: 7.52 (m, 2H, ArH), 7.07 (m, 2H, ArH), 6.84 (s, 1H, thiazole 2-H), 4.51 (bs, 1H, NHCO), 3.98 (m, 2H, piperidine H2a, H6a), 3.97 (m, 1H, CH(CH_3_)_2_), 3.15 (m, 1H, CH), 2.93 (m, 2H, piperidine H2e, H6e), 2.62 (s, 3H, CH_3_), 2.11 (m, 2H, piperidine H3a, H5a), 1.78 (m, 2H, piperidine H3e, H5e), 1.16 (m, 6H, (CH_3_)_2_); ^13^C-NMR (CDCl_3_) δ: 174.11, 168.21, 164.16, 162.50, 160.31, 156.78, 144.35, 130.44, 125.18, 115.39, 110.36, 43.50, 42.42, 40.22, 31.85, 23.25, 12.23; LC-MS m/z: 428.5 (M), 450.3 (M + Na^+^); Elemental Anal. calcd. for C_22_H_25_FN_4_O_2_S: C, 61.66; H, 5.88; N, 13.07; Found: C, 61.72; H, 5.93; N, 13.10.

*N-butyl-4-(4-(3-(4-fluorophenyl)-5-methylisoxazol-4-yl)thiazol-2-yl)piperidine-1-carboxamide* (**4i**). White solid, mp 111.0~111.8 ºC, yield 96%. IR (KBr) ν: 3370 (N-H), 1618 (C=O), 1545, 1460, 1227 (C-F) cm^-1^; ^1^H-NMR (CDCl_3_) δ: 7.49~7.44 (m. 2H, ArH), 7.26 (m, 1H, ArH), 7.12 (m, 1H, ArH), 6.68 (s, 1H, thiazole 2-H), 4.45 (s, 1H, NHCO), 3.99 (d, 2H, piperidine H2a, H6a), 3.24 (m, 2H,CH_2_), 3.13 (m, 1H, CH), 2.95 (m, 2H, piperidine H2e, H6e), 2.72 (s, 3H, CH_3_), 2.08 (m,, 2H, piperidine H3a, H5a), 1.77 (m, 2H, piperidine H3e, H5e), 1.51 (m, 2H, CH_2_), 1.36 (m, 2H, CH_2_), 0.93 (t, *J*=7.2 Hz, 3H, CH_3_); ^13^C-NMR (CDCl_3_) δ: 174.08, 168.30, 164.16, 162.45, 160.30, 156.81, 144.35, 130.45, 125.19, 115.41, 110.16, 43.65, 40.67, 40.15, 32.36, 31.73, 20.02, 13.87, 12.48; LC-MS m/z: 442.5 (M), 464.1 (M + Na^+^); Elemental Anal. calcd. for C_23_H_27_FN_4_O_2_S: C, 62.42; H, 6.15; N, 12.66; Found: C, 62.40; H, 6.20; N, 12.68.

*N-phenyl-4-(4-(3-(4-fluorophenyl)-5-methylisoxazol-4-yl)thiazol-2-yl)piperidine-1-carboxamide* (**4j**). White solid, m.p. 146.6~147.0 ºC, yield 94%. IR (KBr) ν: 3201 (N-H), 1621 (C=O), 1597, 1535, 1228 (C-F) cm^-1^; ^1^HNMR (CDCl_3_) δ: 7.51 (m, 2H, ArH), 7.36 (m, 2H, ArH), 7.26 (m, 2H, ArH), 7.08 (t, *J*=9 Hz, 2H, ArH), 7.02 (m, 1H, ArH), 6.82 (s, 1H, thiazole 2-H), 6.62 (s, 1H, NHCO), 4.15 (d, 2H, piperidine H2a, H6a), 3.20 (m, 1H, CH), 3.05 (m, 2H, piperidine H2e, H6e), 2.60 (s, 3H, CH_3_), 2.14 (m, 2H, piperidine H3a, H5a), 1.83 (m, 2H, piperidine H3e, H5e); ^13^C-NMR (CDCl_3_) δ: 173.86, 168.36, 164.31, 160.47, 154.91, 144.58, 138.98, 130.56, 128.79, 123.05, 119.99, 115.52, 110.43, 43.99, 40.21, 31.98, 12.38; LC-MS m/z: 462.5 (M), 484.4 (M + Na^+^); Elemental Anal. calcd. for C_25_H_23_FN_4_O_2_S: C, 64.92; H, 5.01; N, 12.11; Found: C, 64.87; H, 5.09; N, 12.13.

*N-phenyl-4-(4-(3-(4-fluorophenyl)-5-methylisoxazol-4-yl)thiazol-2-yl)piperidine-1-thiocarboxamide* (**4k**). White solid, mp. 160.3~160.8 ºC, yield 96%. IR (KBr) ν: 3204 (N-H), 1534, 1491, 1226 (C-F), 835 (C=S) cm^-1^; ^1^H-NMR (CDCl_3_) δ: 7.49~7.41 (m, 3H, ArH), 7.32 (m, 2H, ArH), 7.24 (m, 1H, NHCS), 7.16~7.10 (m, 4H, ArH), 6.71 (s, 1H, thiazole 2-H), 4.46 (d, 2H, piperidine H2a, H6a), 3.31 (m, 2H, piperidine H2e, H6e), 3.30 (m, 1H, CH), 2.71 (s, 3H, CH_3_), 2.11 (d, 2H, piperidine H3a, H5a), 1.88 (m, 2H, piperidine H3e, H5e); ^13^C-NMR (CDCl_3_) δ: 182.80, 172.71, 167.71, 161.21, 158.71, 157.25, 144.61, 139.99, 131.61, 129.00, 125.01, 124.34, 122.93, 117.63, 115.75, 113.63, 111.56, 48.97, 39.41, 31.41, 12.72; LC-MS m/z: 478.1 (M), 500.2 (M + Na^+^); Elemental Anal. calcd. for C_25_H_23_FN_4_OS_2_: C, 62.74; H, 4.84; N, 11.71; Found: C, 62.77; H, 4.80; N, 11.73.

*N-isopropyl-4-(4-(3-(2-fluorophenyl)-5-methylisoxazol-4-yl)thiazol-2-yl)piperidine-1-1-carboxamide* (**4l**). White needles, mp 97.1~98.9 ºC, yield 92%. IR (KBr) ν: 3359 (N-H), 1619 (C=O), 1551, 1458, 1224 (C-F) cm^-1^; ^1^H-NMR (CDCl_3_) δ: 7.51~7.45 (m, 2H, ArH), 7.27 (m, 1H, ArH), 7.13 (m, 1H, ArH), 6.68 (s, 1H, thiazole 2-H), 4.25 (s, 1H, NHCO), 3.98 (m, 2H, piperidine H2a, H6a), 3.97 (m, 1H, CH(CH_3_)_2_), 3.15 (m, 1H, CH), 2.93 (m, 2H, piperidine H2e, H6e), 2.72 (s, 3H, CH_3_), 2.09 (m, 2H, piperidine H3a, H5a), 1.77 (m, 2H, piperidine H3e, H5e), 1.16 (m, 6H, (CH_3_)_2_); ^13^C-NMR (CDCl_3_) δ: 173.60, 167.77, 161.23, 158.67, 157.44, 144.81, 131.69, 124.34, 117.69, 116.10, 113.48, 110.67, 43.51, 42.45, 40.30, 31.90, 23.25, 12.43; LC-MS m/z: 428.5 (M), 450.2 (M + Na^+^); Elemental Anal. calcd. for C_22_H_25_FN_4_O_2_S: C, 61.66; H, 5.88; N, 13.07; Found: C, 61.70; H, 5.85; N, 13.05.

*N-butyl-4-(4-(3-(2-fluorophenyl)-5-methylisoxazol-4-yl)thiazol-2-yl)piperidine-1-carboxamide* (**4m**). White solid, mp 107.6~108.7 ºC, yield 94%. IR (KBr) ν: 3370 (N-H), 1618 (C=O), 1545, 1487, 1226 (C-F) cm^-1^; ^1^H-NMR (CDCl_3_) δ: 7.51~7.45 (m, 2H, ArH), 7.25 (m, 1H, ArH), 7.12 (m, 1H, ArH), 6.68 (s, 1H, thiazole 2-H), 4.47 (s, 1H, NHCO), 3.95 (d, 2H, piperidine H2a, H6a), 3.23 (m, 2H, CH_2_), 3.13 (t, *J*=10.8 Hz, 1H, CH), 2.94 (m, 2H, piperidine H2e, H6e), 2.72 (s, 3H, CH_3_), 2.08 (d, 2H, piperidine H3a, H5a), 1.75 (m, 2H, piperidine H3e, H5e), 1.51 (m, 2H, CH_2_), 1.36 (m, 2H, CH_2_), 0.93 (t, *J*=7.2 Hz, 3H, CH_3_); ^13^C-NMR (CDCl_3_) δ: 173.63, 167.76, 161.33, 158.84, 157.54, 144.66, 131.58, 124.35, 117.80, 116.02, 113.46, 110.66, 43.60, 40.67, 40.13, 32.32, 31.85, 20.08, 13.83, 12.74; LC-MS m/z: 442.5 (M), 464.2 (M + Na^+^); Elemental Anal. calcd. for C_23_H_27_FN_4_O_2_S: C, 62.42; H, 6.15; N, 12.66; Found: C, 62.38; H, 6.17; N, 12.63.

*N-phenyl-4-(4-(3-(2-fluorophenyl)-5-methylisoxazol-4-yl)thiazol-2-yl)piperidine-1-carboxamide* (**4n**).

White solid, mp 148~150 ºC, yield 95%. IR (KBr) ν: 3202 (N-H), 1621 (C=O), 1597, 1534, 1226 (C-F) cm^-1^; ^1^H-NMR (CDCl_3_) δ: 7.51~7.45 (m, 2H, ArH), 7.35 (m, 2H, ArH), 7.26~7.23 (m, 2H,ArH), 7.12 (m, 1H, ArH), 7.04 (m, 1H, ArH), 6.71 (s, 1H, thiazole 2-H), 6.43 (s, 1H, NHCO), 4.07 (m, 2H, piperidine H2a, H6a), 3.19 (m, 1H, CH), 3.06 (m, 2H, piperidine H2e, H6e), 2.74 (s, 3H, CH_3_), 2.14 (m, 2H, piperidine H3a, H5a), 1.88 (m, 2H, piperidine H3e, H5e); ^13^C-NMR (CDCl_3_) δ: 174.10, 167.96, 161.16, 159.10, 157.74, 144.57, 131.47, 125.15, 120.13, 117.76, 115.74, 113.46, 110.66, 44.01, 40.31, 31.99, 12.40; LC-MS m/z: 462.5 (M), 484.2 (M + Na^+^); Elemental Anal. calcd. for C_25_H_23_FN_4_O_2_S: C, 64.92; H, 5.01; N, 12.11; Found: C, 64.89; H, 5.07; N, 12.14.

*N-phenyl-4-(4-(3-(2-fluorophenyl)-5-methylisoxazol-4-yl)thiazol-2-yl)piperidine-1-thiocarboxamide* (**4o**). White solid, mp 154.9~156.3 ºC, yield 93%. IR (KBr) ν: 3210 (N-H), 1597, 1535, 1429, 1228 (C-F), 837 (C=S) cm^-1^; ^1^H-NMR (CDCl_3_) δ: 7.51~7.45 (m, 2H, ArH), 7.35 (m, 2H, ArH), 7.32 (s, 1H, NHCS), 7.22 (m, 1H, ArH), 7.16~7.10 (m, 4H, ArH), 6.71 (s, 1H, thiazole 2-H), 4.46 (d, 2H, piperidine H2a, H6a), 3.31 (m, 2H, piperidine H2e, H6e), 3.24 (m, 1H, CH), 2.71 (s, 3H, CH_3_), 2.13 (m, 2H, piperidine H3a, H5a), 1.90 (m, 2H, piperidine H3e, H5e); ^13^C-NMR (CDCl_3_) δ: 183.39, 172.69, 167.37, 161.38, 158.89, 157.34, 144.88, 140.13, 131.69, 129.21, 125.08, 124.37, 122.58, 117.89, 116.03, 113.63, 111.65, 49.18, 39.49, 31.46, 12.77; LC-MS m/z: 478.1 (M), 500.4 (M + Na^+^); Elemental Anal. calcd. for C_25_H_23_FN_4_OS_2_: C, 62.74; H, 4.84; N, 11.71; Found: C, 62.69; H, 4.87; N, 11.77.

## 4. Conclusions and Perspective

In summary, we have synthesized a series of novel 4-(4-(5-methyl-3-arylisoxazol-4-yl)thiazol-2-yl)piperidyl carboxamides and thiocarboxamides. The *in vivo* biological evaluation showed that these compounds had moderate to good herbicidal activities. Since these compounds were designed according to the hit information from virtual screening, and the enzyme activity of one compound also showed the potency targeting D1 protease, it could preliminarily concluded that the bioactivities of these compounds were due to the inhibition of D1 protease.

By comparing the biological activities from the prediction by the Autodock program, the enzyme activity and the *in vivo* assay, there is a direct impression that the *in vivo* biological activity is much lower than expected. This phenomenon is preliminarily attributed to the low bioavailability of these compounds. In fact, these compounds have low solubilities in Tween^®^ 80-water emulsions, as observed in the biological activity test, and their lipophilic clogP values are relatively high, as demonstrated in the Discussion section. Relative high molecular weights perhaps are another issue to cause the low bioavailability. Therefore, it is necessary for a further modification of the lead hit structure and redesign of series of 4-(4-(5-methyl-3-arylisoxazol-4-yl)thiazol-2-yl)piperidyl carboxamides and thiocarboxamides. This work is currently under way in our laboratory.
